# Bazi Bushen Attenuates Skeletal Muscle Decline Associated With Oestrogen Deficiency and Modulates FGFR1/ERK Signalling

**DOI:** 10.1111/jcmm.71407

**Published:** 2026-10-30

**Authors:** Dezhi Huang, Na Xu, Qi Chu, Zhenhua Jia, Yan Wang, Jin Xu

**Affiliations:** ^1^ Key Laboratory of Endocrine Glucose & Lipids Metabolism and Brain Aging, Ministry of Education; Department of Endocrinology Shandong Provincial Hospital Affiliated to Shandong First Medical University Jinan Shandong China; ^2^ Shandong Key Laboratory of Endocrine Metabolism and Aging Jinan Shandong China; ^3^ Shandong Institute of Endocrine and Metabolic Diseases Jinan Shandong China; ^4^ State Key Laboratory for Innovation and Transformation of Luobing Theory Hebei Yiling Hospital Shijiazhuang Hebei China; ^5^ Shandong Provincial Hospital Affiliated to Shandong First Medical University “Chuangxin China” Innovation Base of Stem Cell and Gene Therapy for Endocrine Metabolic Diseases Jinan Shandong China; ^6^ Shandong Provincial Hospital Affiliated to Shandong First Medical University Shandong Engineering Laboratory of Prevention and Control for Endocrine and Metabolic Diseases Jinan Shandong China; ^7^ Shandong Engineering Research Center of Stem Cell and Gene Therapy for Endocrine and Metabolic Diseases Jinan Shandong China

**Keywords:** Bazi Bushen, FGFR1/ERK signalling, oestrogen deficiency, skeletal muscle deterioration

## Abstract

Postmenopausal oestrogen deficiency is associated with skeletal muscle loss and functional decline, thereby increasing the risk of sarcopenia in women. Considering the safety risks linked to hormone replacement therapy (HRT), alternative strategies are needed to attenuate skeletal muscle decline after menopause. A total of 24 female C57BL/6J mice were randomly assigned to four groups (*n* = 6 per group): Sham‐operated (SHAM), ovariectomized (OVX), OVX treated with estradiol valerate (OVX + E2) and OVX treated with Bazi Bushen (OVX + BZBS). Skeletal muscle mass and function were evaluated in four groups. RNA sequencing was applied to explore the molecular mechanisms and key signalling pathways underlying BZBS‐induced muscle protection. We further examined the role of the FGFR1/ERK signalling pathway in mediating the effects of BZBS on myogenic differentiation in C2C12 myoblasts. The results showed that OVX mice had reduced grip strength and skeletal muscle mass, increased fat accumulation, reduced lean mass and impaired metabolic activity. These phenotypic alterations were accompanied by myofiber atrophy, increased collagen staining, endocrine perturbations and elevated expression of p16, p21, Atrogin‐1 and MuRF1. BZBS ameliorated these abnormalities. RNA‐seq suggested that FGF/MAPK signalling may be involved. In D‐gal‐treated C2C12 myoblasts, BZBS reduced Atrogin‐1 and MuRF1 expression while restoring MYHC and MYOG expression. Mechanistically, BZBS suppressed FGFR1/ERK signalling activation and bFGF partially reversed the protective effects of BZBS. Taken together, these findings suggest that FGFR1/ERK signalling may contribute to the protective effects of BZBS. Our findings provide evidence for the beneficial effects of BZBS on skeletal muscle deterioration associated with oestrogen deficiency.

## 1 Introduction

Skeletal muscle is a key organ for maintaining locomotor capacity, energy metabolism and healthy aging [1]. As people age, skeletal muscle strength, mass and function progressively decline, which may ultimately contribute to sarcopenia [2]. Reduced muscle strength is identified as a central criterion for sarcopenia [3]. As global population aging accelerates, sarcopenia has become an important public health concern. A global meta‐analysis reported that the prevalence of sarcopenia among individuals aged 60 years or older is approximately 10%–27% [4]. Previous epidemiological studies have shown that the age at natural menopause in women is typically 50–51 years [5]. In women, the decline in oestrogen levels after menopause is closely associated with abnormalities in bone metabolism and also affects skeletal muscle mass, strength and contractile function, which may further increase the risk of sarcopenia [6, 7, 8].

Previous studies have shown that hormone replacement therapy (HRT) may help maintain or improve muscle mass and strength. Oestrogen may facilitate recovery following muscle atrophy and injury, rendering the hormone especially critical for maintaining skeletal muscle health in postmenopausal women [9, 10]. However, HRT is not suitable for all postmenopausal women, as its benefits and potential risks vary according to individual clinical characteristics. Factors such as age, contraindications, the timing of treatment initiation and the risk of thromboembolic events or stroke should therefore be carefully considered [11]. Accordingly, alternative strategies are needed to attenuate skeletal muscle decline after menopause.

Traditional Chinese medicine (TCM) has a long history of clinical use and has attracted increasing interest as a potential approach to aging‐related disorders and chronic diseases [12, 13]. Previous studies have shown that herbal medicines and their bioactive components, such as Chinese yam, quercetin and black ginseng extract, can promote muscle protein synthesis and inhibit muscle degradation by regulating the Akt/mTOR signalling pathway [14, 15, 16]. Clinical evidence also suggests that TCM‐based interventions may improve sarcopenia‐related outcomes, particularly in older adults [17]. These findings indicate that TCM may provide a potential strategy for maintaining skeletal muscle mass and function during aging. Bazi Bushen (BZBS) is a traditional Chinese herbal formula used to alleviate age‐related functional decline [18]. Previous clinical studies have shown that BZBS has favourable efficacy and safety in individuals with premature aging [19]. However, its effects on skeletal muscle decline associated with postmenopausal oestrogen deficiency remain unclear.

In this study, an ovariectomized (OVX) mouse model was used to mimic postmenopausal oestrogen deficiency, with E2 treatment as a positive reference [20]. BZBS was administered to systematically evaluate the effects on muscle mass and function. We further used a D‐gal‐treated C2C12 myoblast model to examine the cellular effects of BZBS and explore the potential signalling mechanisms [21]. This study aimed to provide experimental evidence for the potential use of BZBS against skeletal muscle deterioration associated with oestrogen deficiency.

## 2 Methods

### 2.1 Preparation and Quality Control (QC) of BZBS

BZBS was obtained from Shijiazhuang Yiling Pharmaceutical Co. Ltd. (Shijiazhuang, China). Its preparation and quality control have been previously described, including UPLC‐based fingerprint analysis demonstrating batch consistency and identifying representative compounds [18].

### 2.2 Animals

A total of 24 female C57BL/6J mice were purchased from Beijing Vital River Laboratory Animal Technology Co. Ltd. (Beijing, China). At the time of purchase, the mice were 8 weeks old and weighed 18–22 g. All mice were housed at the Experimental Animal Center of Shandong Provincial Hospital Affiliated to Shandong First Medical University under controlled environmental conditions, including a room temperature of 20°C–22°C, a 12 h light/12 h dark cycle and free access to food and water. Mice were maintained under standard conditions until 28 weeks of age and were then randomly assigned to four groups: the sham surgery group (SHAM), the OVX model group (OVX), the E2 treatment group (OVX + E2) and the BZBS treatment group (OVX + BZBS). Each group contained six mice. All six mice were used for body composition, skeletal muscle wet weight and grip strength measurements, whereas three mice per group were randomly selected for indirect calorimetry, serum ELISA and tissue‐based analyses according to the original experimental design. Each mouse was considered an independent biological replicate. Mice in the SHAM group underwent sham surgery, whereas mice in the other three groups underwent bilateral ovariectomy to mimic postmenopausal oestrogen deficiency. Interventions were started 1 week after surgery. Mice in the OVX + E2 group were fed a diet containing 0.26% Progynova (estradiol valerate, Bayer, Germany). This regimen was selected according to previous studies to restore systemic oestrogen levels [22]. Mice in the OVX + BZBS group were fed a diet containing BZBS at a dose of 2 g/kg/day [23]. Mice in the SHAM and OVX groups received a standard maintenance diet. All mice were maintained under the same conditions until 50 weeks of age. Body weight was recorded regularly throughout the experiment (Figure 1A). Samples were uniformly coded and investigators were blinded to group allocation during data acquisition and quantitative analyses.

**FIGURE 1:**
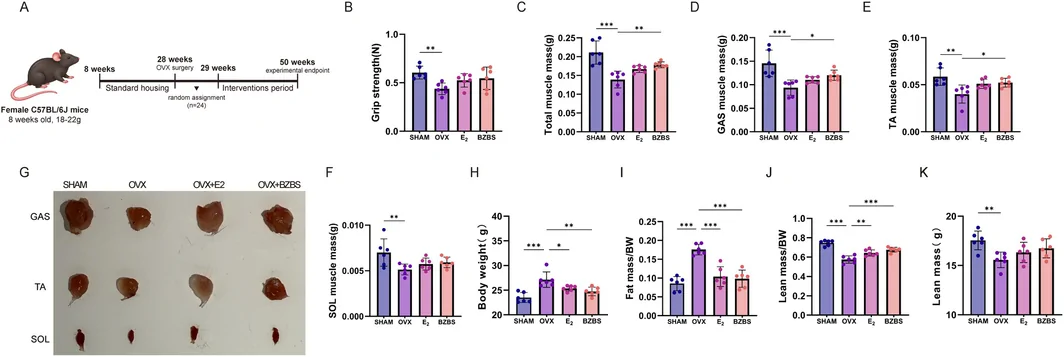
BZBS improves skeletal muscle function, muscle mass and body composition in OVX mice. (A) Experimental timeline and grouping strategy. Female C57BL/6J mice were randomly assigned to the SHAM, OVX, OVX + E2 and OVX + BZBS groups. (B) Forelimb grip strength in each group. (C–F) Quantification of total skeletal muscle mass, gastrocnemius (GAS) mass, soleus (SOL) mass and tibialis anterior (TA) mass. (G) Representative gross images of GAS, TA and SOL muscles from each group. (H–K) Body weight, fat mass/body weight ratio, lean mass/body weight ratio and lean mass measured by nuclear magnetic resonance. Data are presented as the mean ± SD, with each data point representing one independent mouse (*n* = 6 per group). Statistical comparisons were performed using one‐way analysis of variance (ANOVA), followed by Tukey's post hoc test to determine significance. *p < 0.05, **p < 0.01, ***p < 0.001.

At the experimental endpoint, mice were anaesthetised by intraperitoneal injection of tribromoethanol (Avertin, 290 mg/kg) [24], and blood samples were collected. The mice were then euthanized by cervical dislocation and relevant tissues were rapidly isolated for subsequent analyses. All animal procedures were conducted in accordance with institutional guidelines and were approved by the Ethics Committee of Shandong Provincial Hospital Affiliated to Shandong First Medical University (approval no. SWYX 2024–166).

### 2.3 Statistical Analysis

Quantitative data are presented as the mean ± standard deviation (SD). Time‐course data from indirect calorimetry are presented as the mean ± standard error of the mean (SEM). Each mouse was considered an independent biological replicate. Normality and homogeneity of variance were assessed using the Shapiro–Wilk and Brown–Forsythe tests, respectively. Data meeting the assumptions of normality and homogeneity of variance were analysed using one‐way analysis of variance (ANOVA), followed by Tukey's multiple comparisons test. For datasets that did not meet the normality assumption, differences among multiple groups were analysed using the Kruskal–Wallis test, followed by Dunn's multiple comparisons test. All statistical analyses were performed using GraphPad Prism 10 (GraphPad Software, San Diego, CA, USA). A two‐sided *p* < 0.05 was considered statistically significant. Statistical significance was denoted as follows: *p < 0.05, **p < 0.01 and ***p < 0.001. Exact *p* values for the principal comparisons are provided in Table S2.

Additional methods are described in the Supporting Information and Methods and the primer sequences used for qPCR are provided in Table S1.

## 3 Results

### 3.1 BZBS Ameliorates Skeletal Muscle Dysfunction, Body Composition Imbalance and Reduced Energy Metabolism in OVX Mice

To systematically evaluate skeletal muscle function in OVX mice, we first performed a forelimb grip strength test. Compared with SHAM mice of the same age, OVX mice showed a marked reduction in grip strength, indicating impaired muscle function after ovariectomy. Higher mean grip strength values were observed after E2 and BZBS treatment (Figure 1B). Subsequent analysis of three skeletal muscles, including the gastrocnemius, tibialis anterior and soleus muscles, further showed that total skeletal muscle weight was significantly reduced in OVX mice. In particular, gastrocnemius (GAS) wet weight was markedly decreased, suggesting skeletal muscle atrophy after ovariectomy. Treatment with E2 or BZBS attenuated these decreases in muscle weight to varying degrees (Figure 1C–G).

Body composition analysis by NMR showed that OVX mice had increased body weight and fat percentage, together with significant decreases in lean mass and lean mass percentage. These findings indicated an imbalance in body composition after ovariectomy, characterized by fat accumulation and reduced relative lean mass. Treatment with E2 or BZBS improved body composition, as reflected by lower body weight and fat percentage and higher lean mass percentage (Figure 1H–K).

To further determine whether these changes were accompanied by abnormalities in whole‐body energy metabolism, metabolic parameters were analysed over a 24 h period using a metabolic cage system. Differences among groups were mainly observed during the dark phase, whereas no obvious differences were detected during the light phase. Because mice are primarily active and consume food during the dark phase, we further quantified metabolic changes during this period from 19:00 to 7:00. Compared with SHAM mice, OVX mice showed decreasing trends in oxygen consumption (VO_2_), carbon dioxide production (VCO_2_), activity and energy expenditure (EE) during the dark phase, suggesting reduced overall metabolic activity after ovariectomy. Treatment with E2 or BZBS increased these metabolic parameters to varying degrees, suggesting partial improvement in dark‐phase metabolic activity (Figure 2A–H). In addition, the daily fluctuation in respiratory exchange ratio (RER) was markedly blunted in OVX mice compared with the other three groups, suggesting an altered diurnal pattern of substrate utilization. Treatment with E2 or BZBS partially restored the diurnal variation in RER (Figure 2I).

**FIGURE 2:**
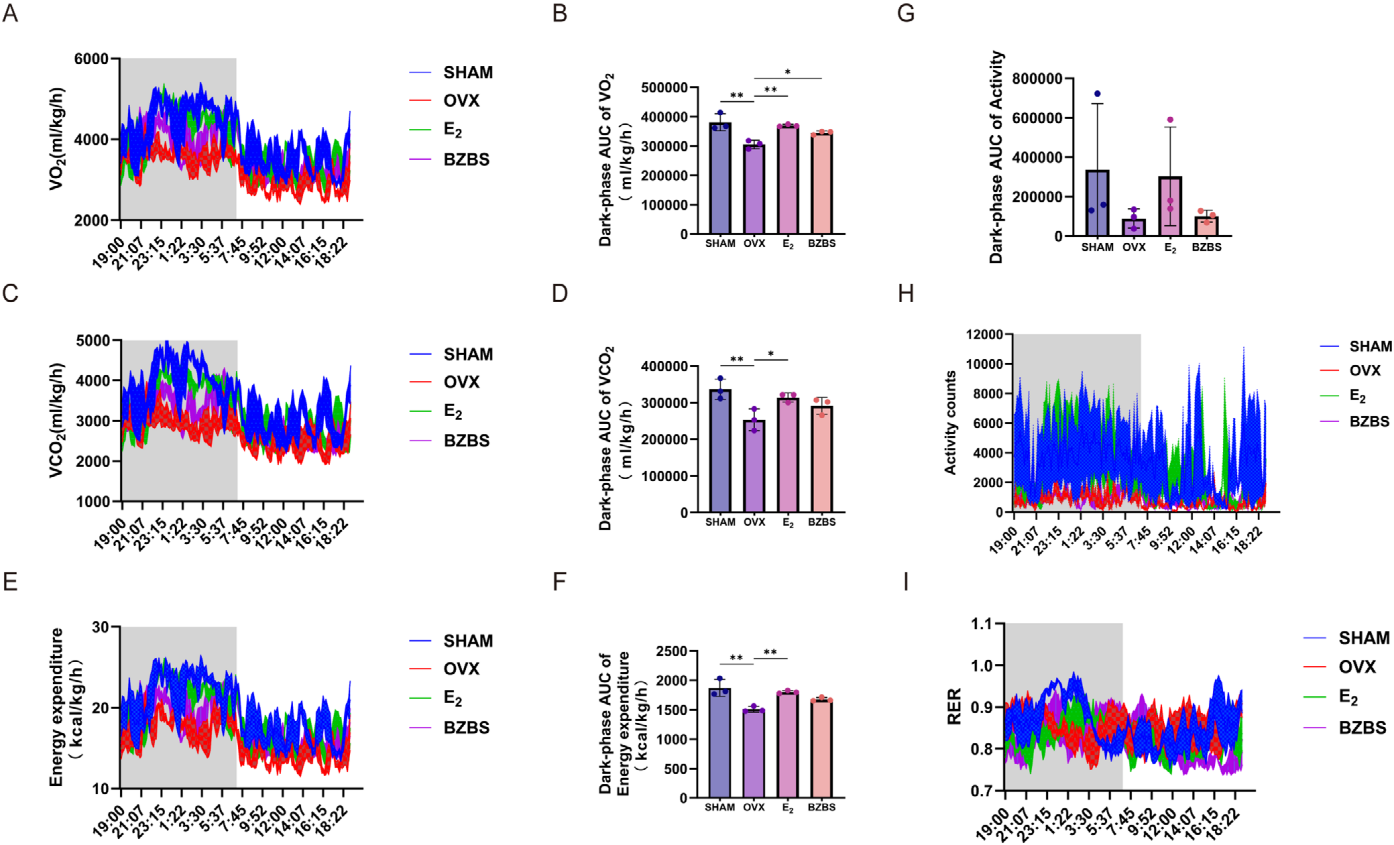
BZBS improves dark‐phase metabolic activity in OVX mice. (A, B) 24 h oxygen consumption (VO_2_) curve and dark phase AUC quantification. (C, D) 24 h carbon dioxide production (VCO_2_) curve and dark phase AUC quantification. (E, F) 24 h energy expenditure (EE) curve and dark phase AUC quantification. (G, H) 24 h activity curve and dark phase AUC quantification. (I) 24 h respiratory exchange ratio (RER) curve. Time‐course data are presented as the mean ± SEM, whereas the corresponding dark‐phase AUC values are presented as the mean ± SD, with each data point representing one independent mouse (*n* = 3 per group). Grey shading indicates the dark phase. Statistical comparisons were performed using one‐way analysis of variance (ANOVA), followed by Tukey's post hoc test to determine significance. *p < 0.05, **p < 0.01.

Overall, BZBS improved skeletal muscle function, muscle mass, body composition and dark‐phase metabolic activity in OVX mice, with changes broadly similar in direction to those observed after E2 treatment.

### 3.2 BZBS Attenuates Myofiber Atrophy and Collagen Deposition in OVX Mice

To further determine the effects of ovariectomy on skeletal muscle structure, haematoxylin and eosin (HE) staining was performed on GAS tissue sections. Compared with SHAM mice, OVX mice showed marked atrophic changes in the GAS, including disorganized myofiber arrangement, heterogeneous myofiber size and widened interfascicular spaces. After treatment with E2 or BZBS, myofiber arrangement in the GAS was markedly improved, the tissue architecture became more compact and the cross‐sectional morphology and size of myofibers became more uniform (Figure 3A). Further quantitative analysis showed that the mean cross‐sectional area (MCSA) of GAS myofibers was significantly reduced in OVX mice, indicating marked myofiber atrophy after ovariectomy. E2 and BZBS treatment significantly increased MCSA, suggesting that both treatments alleviated myofiber atrophy induced by ovariectomy (Figure 3B). Masson staining further showed greater collagen deposition in the GAS of OVX mice than in SHAM mice. In contrast, less collagen staining was observed after E2 or BZBS treatment (Figure 3A). Taken together, these results indicate that BZBS ameliorates myofiber atrophy and fibrotic changes after ovariectomy.

**FIGURE 3:**
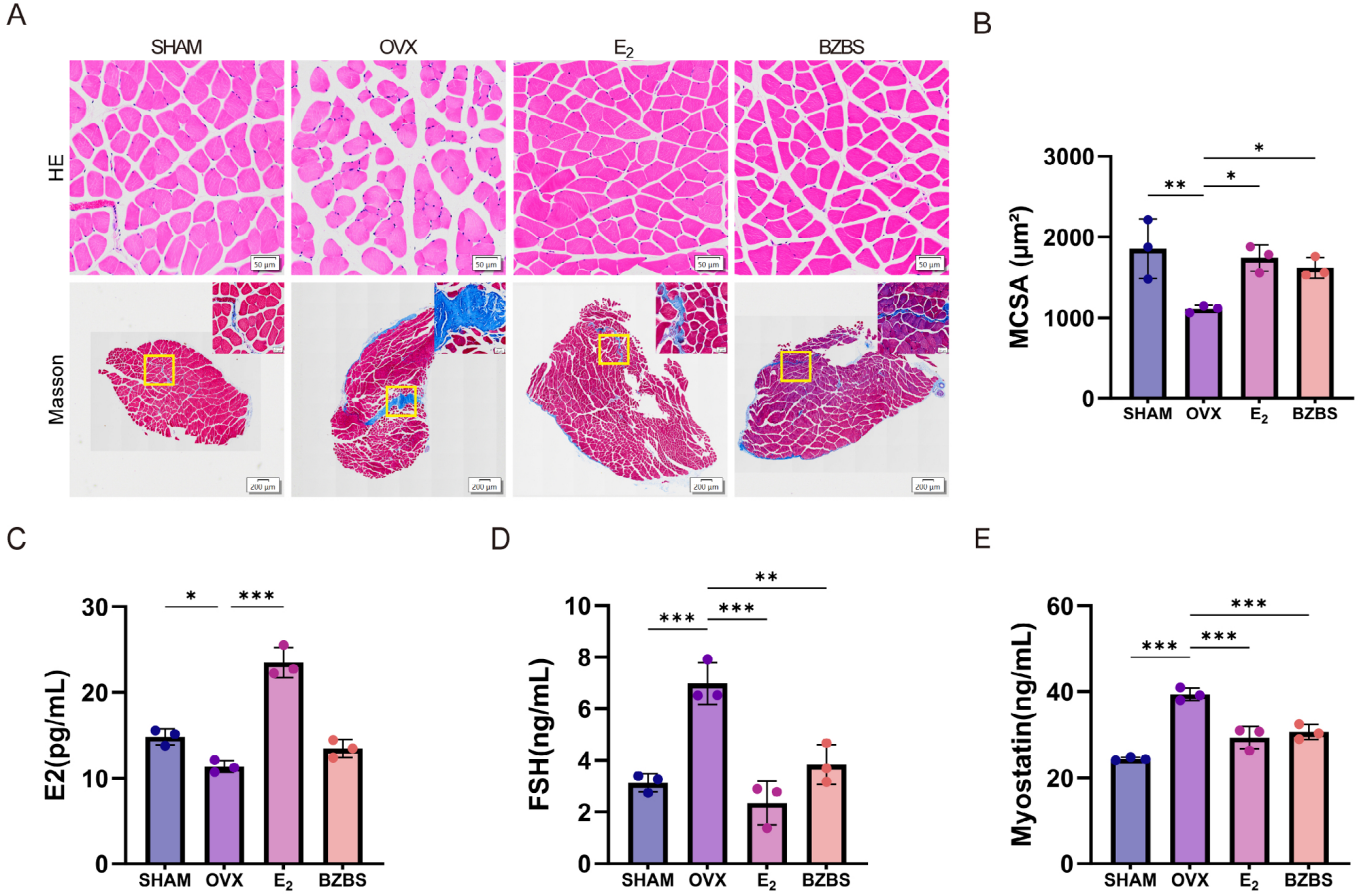
BZBS alleviates GAS myofiber atrophy, collagen deposition and serum myostatin elevation in OVX mice. (A) Representative haematoxylin and eosin (HE) and Masson staining images of GAS sections. HE staining was used to assess myofiber morphology and Masson staining was used to evaluate collagen deposition. (B) Quantification of mean cross‐sectional area (MCSA) of GAS myofibers. (C–E) Serum levels of E2, FSH and myostatin measured by ELISA. Data are presented as the mean ± SD, with each data point representing one independent mouse (*n* = 3 per group). Statistical comparisons were performed using one‐way analysis of variance (ANOVA), followed by Tukey's post hoc test to determine significance. *p < 0.05, **p < 0.01, ***p < 0.001.

### 3.3 BZBS Modulates Serum E2 and FSH Levels and Reduces Serum Myostatin in OVX Mice

Serum levels of E2, FSH and myostatin were then measured by ELISA. Compared with OVX mice, E2 supplementation significantly increased serum E2 levels that had been reduced by ovariectomy, whereas BZBS showed only a tendency toward increased serum E2. In addition, serum FSH and myostatin levels were markedly elevated in OVX mice, while treatment with E2 or BZBS significantly reduced both FSH and myostatin levels (Figure 3C–E). Therefore, BZBS not only improved local skeletal muscle structure but may also help counteract the inhibition of muscle growth under oestrogen deficiency, possibly through modulation of endocrine imbalance after ovariectomy.

### 3.4 BZBS Reduces the Expression of Markers Associated With Senescence and Atrophy in OVX Mice

To further determine whether skeletal muscle structural damage after ovariectomy was accompanied by changes in molecules associated with senescence and atrophy, immunohistochemical staining was performed to detect p16 and p21 expression in GAS tissues from each group. Compared with SHAM mice, OVX mice showed significantly increased positive staining for p16 and p21 in the GAS, indicating elevated expression of senescence markers in skeletal muscle after ovariectomy. Treatment with E2 or BZBS significantly reduced positive staining for p16 and p21 (Figure 4A–C). Western blot analysis further confirmed these findings. The levels of senescence markers, including p16 and p21 and atrophy markers, including Atrogin‐1 and MuRF1, were significantly increased in skeletal muscle after ovariectomy. Treatment with E2 or BZBS suppressed the abnormal increases in p16, p21, Atrogin‐1 and MuRF1 to varying degrees (Figure 4D–F). These results indicate that BZBS suppresses the elevation of senescence markers induced by ovariectomy in skeletal muscle and reduces the abnormal expression of atrophy markers, which may contribute to its protective effects against skeletal muscle injury after ovariectomy.

**FIGURE 4:**
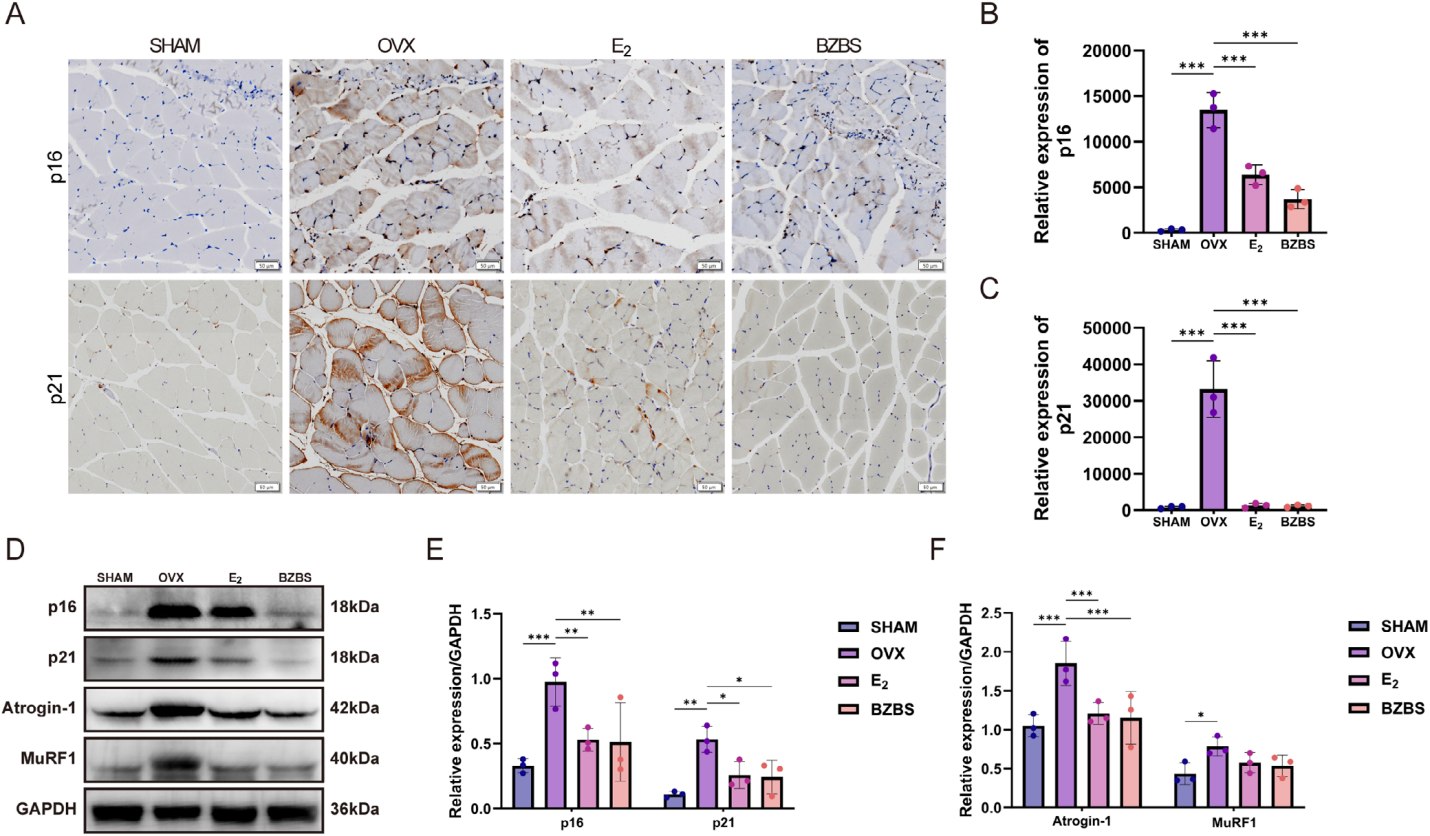
BZBS reduces senescence and atrophy‐related molecular abnormalities in GAS from OVX mice. (A) Representative immunohistochemical staining images of p16 and p21 in GAS sections. (B–C) Quantification of p16 and p21 positive staining areas. (D) Representative Western blot bands of p16, p21, Atrogin‐1 and MuRF1 in GAS tissues. (E–F) Quantification of p16, p21, Atrogin‐1 and MuRF1 protein expression normalized to GAPDH. Data are presented as the mean ± SD, with each data point representing one independent mouse (*n* = 3 per group). Statistical comparisons were performed using one‐way analysis of variance (ANOVA), followed by Tukey's post hoc test to determine significance. *p < 0.05, **p < 0.01, ***p < 0.001.

### 3.5 RNA‐Seq Analysis Suggests a Role for FGF Responses and MAPK Signalling in the Effects of BZBS

Based on the above observations, RNA‐seq analysis was performed on GAS samples from the SHAM, OVX and OVX + BZBS groups using an Illumina sequencing platform to explore transcriptional changes associated with ovariectomy and BZBS treatment. Differentially expressed genes (DEGs) were defined as genes with *p* ≤ 0.05 and |log_2_FC| ≥ 1. Heatmap analysis and principal component analysis (PCA) showed that gene expression was broadly altered in the OVX group compared with the SHAM group and that BZBS treatment partly reshaped this altered transcriptional profile (Figure 5A,B). A total of 1095 genes were differentially expressed between the OVX and SHAM groups, whereas 699 genes were differentially expressed between the OVX + BZBS and OVX groups. Across these two comparisons, 256 overlapping DEGs were identified (Figure 5C). GO and KEGG enrichment analyses were then performed on these 256 overlapping genes. GO biological process analysis showed that these genes were mainly enriched in biological processes related to FGF responses, muscle adaptation, regulation of apoptosis, connective tissue development, autophagy and steroid hormone responses. Representative terms included response to fibroblast growth factor, cellular response to fibroblast growth factor stimulus, muscle adaptation, macroautophagy and cellular response to steroid hormone stimulus. The top 30 GO‐BP enrichment results are provided in Table S3. Among these enriched processes, terms related to FGF responses were prominently enriched (Figure 5D). KEGG pathway enrichment analysis further showed that these overlapping genes were involved in several pathways related to the regulation of muscle homeostasis, including the MAPK signalling pathway, mTOR signalling pathway, FoxO signalling pathway, autophagy and calcium signalling pathway (Figure 5E). Given the enrichment of FGF‐related biological processes in GO analysis and MAPK signalling in KEGG analysis, candidate genes related to these processes, together with genes associated with muscle atrophy, were selected for further validation.

**FIGURE 5:**
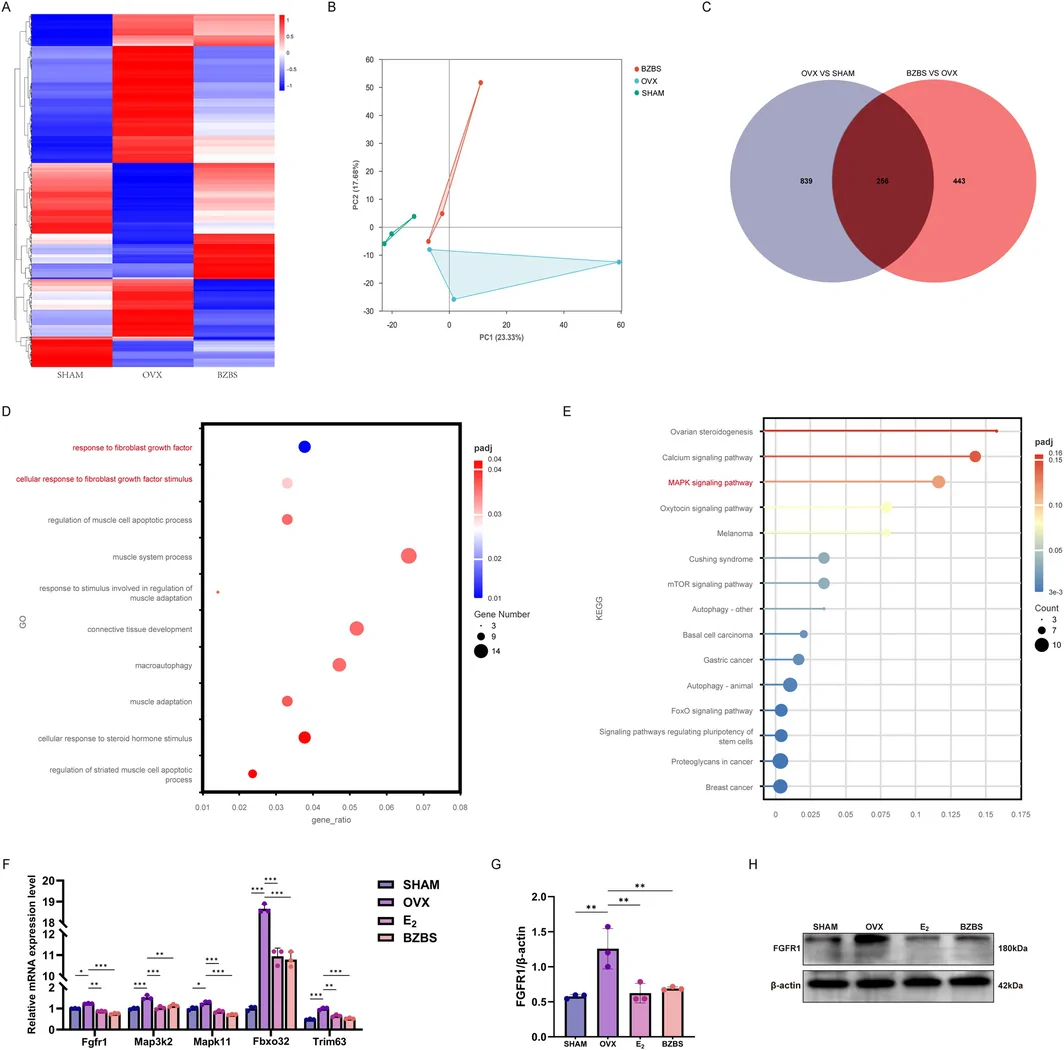
BZBS regulates candidate molecules related to FGF responses, MAPK signalling and muscle atrophy in OVX mice. (A) Heatmap showing differentially expressed genes among SHAM, OVX and OVX + BZBS groups. (B) Principal component analysis (PCA) of RNA‐seq profiles from GAS tissues. (C) Venn diagram showing overlapping differentially expressed genes between OVX vs. SHAM and OVX + BZBS vs. OVX comparisons. (D) GO‐BP enrichment analysis of the 256 overlapping differentially expressed genes. (E) KEGG pathway enrichment analysis of the overlapping differentially expressed genes. (F) RT‐qPCR validation of the mRNA expression of *Fgfr1*, *Map3k2*, *Mapk11*, *Fbxo32* and *Trim63* in GAS tissues. (G–H) Western blot detection and quantification of total FGFR1 protein expression normalized to GAPDH. Data are presented as the mean ± SD, with each data point representing one independent mouse (*n* = 3 per group). Statistical comparisons were performed using one‐way analysis of variance (ANOVA), followed by Tukey's post hoc test to determine significance. *p < 0.05, **p < 0.01, ***p < 0.001.

### 3.6 BZBS Regulates Candidate Molecules Related to FGF Responses, MAPK Signalling and Muscle Atrophy in OVX Mice

Based on the enrichment analysis results, qPCR was performed to further validate relevant candidate genes in GAS tissues. Compared with the SHAM group, the mRNA levels of *Fgfr1*, *Map3k2*, *Mapk11*, *Fbxo32* and *Trim63* were significantly increased in the GAS of OVX mice. Treatment with E2 or BZBS reduced the abnormal increases in the expression of these genes to varying degrees (Figure 5F). Among these genes, *Fbxo32* and *Trim63* encode Atrogin‐1 and MuRF1, respectively. Both proteins are well‐recognized markers of muscle atrophy and the increased mRNA levels of *Fbxo32* and *Trim63* were consistent with the elevated protein levels of Atrogin‐1 and MuRF1 described above (Figure 4D,F). Further Western blot analysis showed that FGFR1 protein expression was markedly increased in the GAS of OVX mice, whereas treatment with E2 or BZBS reduced FGFR1 protein levels (Figure 5G,H). These results suggest that BZBS modulates changes related to FGF responses, MAPK signalling and muscle atrophy after ovariectomy, including changes in *Fgfr1* mRNA and FGFR1 protein levels.

### 3.7 BZBS Improves Myogenic Differentiation in D‐Gal‐Treated C2C12 Cells With Possible Involvement of FGFR1/ERK Signalling

To further determine whether BZBS could directly affect myoblasts, BZBS was added to the culture medium of C2C12 cells. BZBS was first tested at six concentrations, including 0, 20, 100, 200, 500 and 1000 μg/mL and cell viability was assessed using the CCK‐8 assay to determine an appropriate working concentration. BZBS showed no obvious cytotoxicity within the tested concentration range. At 100 μg/mL, BZBS did not materially alter the CCK‐8 signal compared with the control. This concentration was therefore selected for subsequent differentiation experiments to minimize the potential influence of altered cell viability (Figure 6A).

**FIGURE 6:**
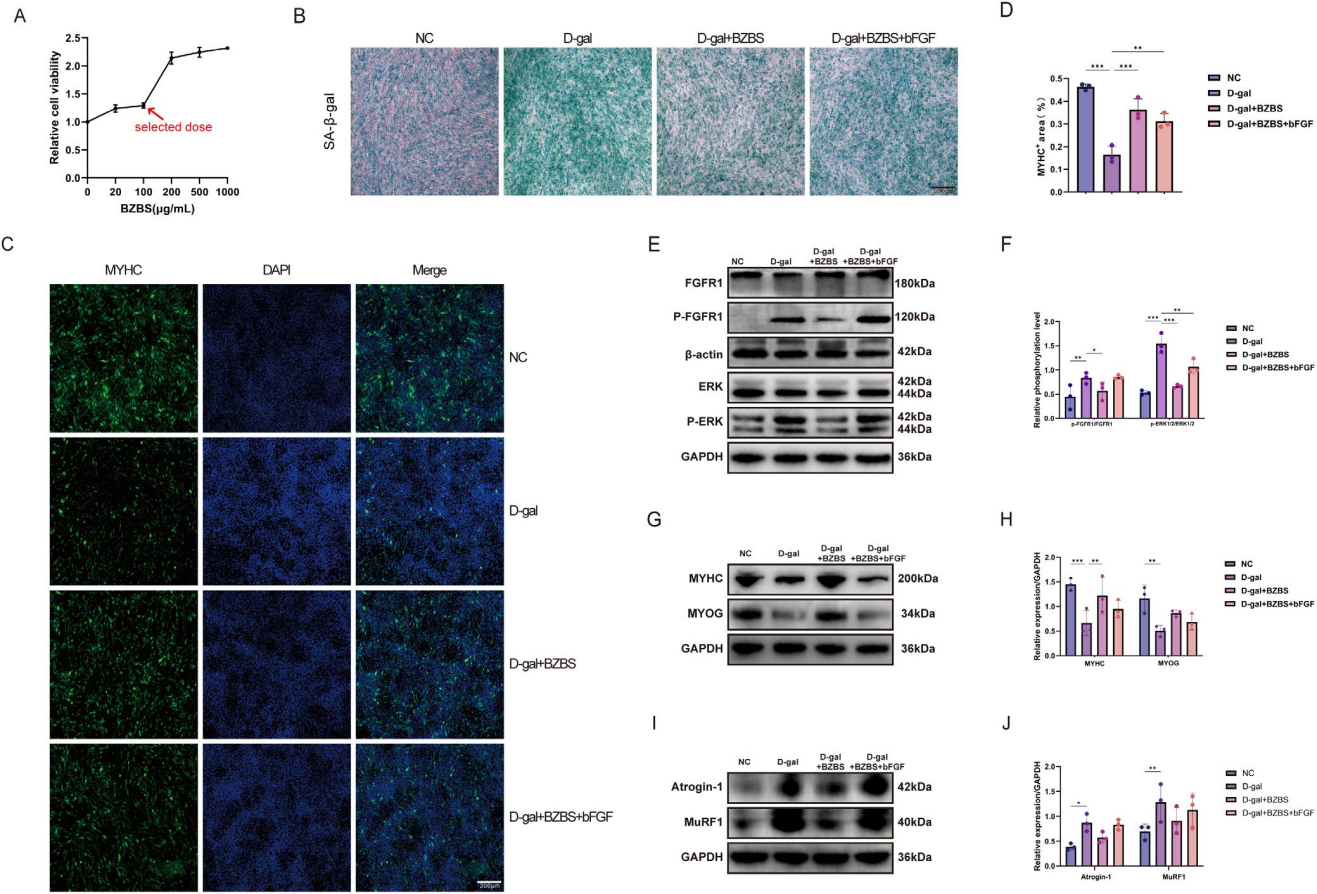
BZBS improves myogenic differentiation in D‐gal‐treated C2C12 cells with possible involvement of FGFR1/ERK signalling. (A) Effects of different concentrations of BZBS on C2C12 cell viability assessed by CCK‐8 assay. The selected dose for subsequent experiments is indicated. (B) Representative SA‐β‐gal staining images of C2C12 cells. (C, D) Representative MYHC immunofluorescence images and quantification of MYHC positive area. (E, F) Representative Western blot bands and quantitative analysis of FGFR1, p‐FGFR1, ERK and p‐ERK in C2C12 cells. The activation levels were evaluated by p‐FGFR1/FGFR1 and p‐ERK/ERK ratios. (G, H) Western blot analysis and quantification of MYHC and MYOG protein expression normalized to GAPDH. (I–J) Western blot analysis and quantification of Atrogin‐1 and MuRF1 protein expression normalized to GAPDH. Data are presented as the mean ± SD, with each data point representing one independent experiment (*n* = 3 per group). Statistical comparisons were performed using one‐way analysis of variance (ANOVA), followed by Tukey's post hoc test to determine significance. *p < 0.05, **p < 0.01, ***p < 0.001.

D‐gal was then used to induce cellular injury accompanied by features of senescence in C2C12 cells and cellular senescence was assessed by SA‐β‐galactosidase staining. Representative images showed increased SA‐β‐gal‐positive staining after D‐gal treatment compared with the NC group. This staining was less prominent after BZBS treatment, whereas the addition of bFGF partially attenuated the effect of BZBS (Figure 6B).

To evaluate the differentiation capacity of C2C12 cells, MYHC immunofluorescence staining was performed. D‐gal treatment significantly reduced myotube formation, whereas BZBS treatment increased the formation of myotubes positive for MYHC. After combined treatment with bFGF, the protective effect of BZBS against impaired differentiation induced by D‐gal was weakened (Figure 6C,D). These findings suggest that BZBS may improve impaired myogenic differentiation induced by D‐gal and that signalling related to FGF may be involved in this effect.

To explore the underlying molecular mechanism, we further examined FGFR1 phosphorylation and ERK activation. Western blot analysis showed that D‐gal increased the levels of phosphorylated FGFR1 (p‐FGFR1) and phosphorylated ERK (p‐ERK), whereas total FGFR1 and total ERK levels were not markedly changed. These findings suggest that D‐gal induced abnormal FGFR1 phosphorylation and ERK activation. BZBS reduced the increases in p‐FGFR1 and p‐ERK caused by D‐gal, suggesting that BZBS attenuated these abnormal changes. After combined treatment with bFGF, p‐FGFR1 and p‐ERK levels increased again, suggesting that bFGF reactivated this signalling axis and thereby partly counteracted the effect of BZBS (Figure 6E,F).

Meanwhile, D‐gal significantly reduced the expression of the myogenic differentiation markers MYOG and MYHC and increased the expression of proteins associated with atrophy, including Atrogin‐1 and MuRF1. BZBS treatment partly restored MYOG and MYHC expression and reduced Atrogin‐1 and MuRF1 levels. However, after combined treatment with bFGF, the effects of BZBS on restoring myogenic differentiation markers and suppressing proteins associated with atrophy were partly weakened (Figure 6G–J).

Taken together, these results indicate that BZBS alleviates impaired myogenic differentiation and molecular changes associated with atrophy induced by D‐gal, possibly by suppressing abnormal FGFR1 phosphorylation and ERK activation. These findings further suggest that FGFR1/ERK signalling may be involved in the regulatory effects of BZBS on myogenic differentiation.

## 4 Discussion

In this study, OVX mice were used to model skeletal muscle changes associated with postmenopausal oestrogen deficiency, with E2 serving as a positive reference treatment. Ovariectomy reduced muscle strength and mass, increased fat accumulation, lowered relative lean mass, decreased metabolic activity during the dark phase and was accompanied by myofiber atrophy and increased expression of markers related to senescence and atrophy. BZBS ameliorated most of these pathological alterations, with effects generally similar to those of E2. RNA sequencing and subsequent validation suggested the involvement of FGF responses and MAPK signalling. BZBS improved myogenic differentiation and suppressed FGFR1 phosphorylation and ERK activation in D‐gal‐treated C2C12 cells, whereas bFGF partially weakened these effects. Overall, BZBS alleviated skeletal muscle impairment associated with oestrogen deficiency, potentially exerting its protective actions through suppression of the FGFR1/ERK signalling pathway.

In this study, OVX mice showed not only reduced grip strength and decreased skeletal muscle weight, but also increased fat accumulation, reduced relative lean mass and reduced whole body metabolic activity during the dark phase. Previous studies have suggested that reduced muscle strength is a core feature in the development and progression of sarcopenia and is often an earlier and more sensitive indicator than the loss of muscle mass alone [3]. Therefore, the reduction in grip strength observed in OVX mice suggests functional impairment of skeletal muscle. The accompanying reduction in skeletal muscle wet weight further supported the presence of muscle atrophy. Notably, changes in muscle wet weight differed among muscle groups in the present study. Mashouri et al. reported that the effects of oestrogen on skeletal muscle may vary according to muscle type and fibre composition. Therefore, the absence of significant changes in the wet weight of the TA and SOL muscles does not necessarily indicate that these muscles were unaffected by ovariectomy [25]. These abnormalities were improved to varying degrees after E2 supplementation, consistent with findings from previous animal studies and reviews. Earlier studies have shown that oestrogen deficiency can induce skeletal muscle atrophy and reduce muscle strength, whereas HRT can improve muscle mass and function and may also ameliorate unfavourable changes in body composition and metabolism in OVX animals [10]. Previous studies have also noted that postmenopausal oestrogen deficiency can exacerbate skeletal muscle deterioration with aging, including losses in muscle mass and declines in muscle quality and contractile function [26, 27]. BZBS also exerted beneficial effects similar in direction to those of E2, suggesting that BZBS may protect skeletal muscle under conditions of oestrogen deficiency.

This study further showed that oestrogen deficiency after ovariectomy was associated with myofiber atrophy and increased collagen staining, with concomitant increases in p16/p21 and Atrogin‐1/MuRF1 expression in skeletal muscle. Previous studies in animal models of ovarian function loss have shown that oestrogen deficiency can reduce skeletal muscle mass, increase the abnormal expression of molecules associated with atrophy, such as Atrogin‐1 and MuRF1 and elevate serum myostatin levels. In contrast, HRT can improve these abnormalities to some extent [28, 29]. Myostatin is a well established negative regulator of muscle growth and previous studies have suggested that reduced myostatin expression is associated with enhanced muscle anabolism and recovery of muscle mass and function [30, 31, 32]. Consistent with these findings, OVX mice in the present study showed reduced serum E2 and increased myostatin, accompanied by decreased skeletal muscle wet weight, reduced MCSA and increased Atrogin‐1/MuRF1 expression. These results suggest that skeletal muscle under oestrogen deficient conditions may be in a state of more pronounced growth inhibition and atrophy. In addition, increased p16/p21 expression was observed, indicating that skeletal muscle injury after ovariectomy was also accompanied by elevated levels of proteins associated with senescence [33]. Both E2 and BZBS improved these histological abnormalities and molecular changes to varying degrees. Interestingly, serum E2 levels were significantly increased after oestrogen supplementation, whereas BZBS only modestly increased serum E2. This finding suggests that the protective effects of BZBS on skeletal muscle may not depend solely on increased circulating E2 levels. In other words, although the protective effects of BZBS on skeletal muscle were directionally consistent with those of E2, its mechanism of action may differ from direct hormone supplementation.

To further investigate the potential molecular mechanisms by which BZBS ameliorates skeletal muscle injury, RNA‐seq was performed on GAS tissues from the SHAM, OVX and OVX + BZBS groups to identify differentially expressed genes (DEGs), followed by GO and KEGG enrichment analyses of the overlapping DEGs. Earlier studies have shown that FGF signalling can promote myoblast proliferation, satellite cell activation and tissue regeneration during the early phase of injury repair [34, 35, 36]. However, recent studies have also indicated that sustained ERK activation mediated by FGF signalling during myogenic differentiation may inhibit terminal differentiation and myotube formation in C2C12 cells [37, 38, 39]. Consistent with these findings, GO analysis in the present study showed that processes related to FGF responses were prominently enriched in the setting of skeletal muscle injury after ovariectomy and BZBS intervention. KEGG analysis further suggested the potential involvement of the MAPK signalling pathway. These results suggest that the transcriptional changes associated with oestrogen deficiency and BZBS intervention may involve alterations in FGF responses and processes related to the MAPK pathway. Therefore, *Fgfr1*, which was present in the gene set enriched for the MAPK signalling pathway, was selected for further validation together with *Map3k2* and *Mapk11*, two genes related to the MAPK cascade and *Fbxo32* and *Trim63*, two effector genes associated with muscle atrophy. *Fgfr1* was therefore selected as a candidate link between FGF‐related responses and downstream MAPK signalling [40].

In OVX mice, we observed not only skeletal muscle atrophy but also abnormal increases in p16/p21 expression in the GAS, suggesting that skeletal muscle injury induced by oestrogen deficiency was accompanied by changes in molecules associated with senescence [41]. Therefore, we further used a D‐gal‐induced C2C12 myoblast injury model with senescence features to investigate whether BZBS could directly affect injured myoblasts and to further explore the potential cellular mechanism. SA‐β‐galactosidase is one of the most widely used markers of cellular senescence [42]. Previous studies have shown that D‐gal can mimic several features of natural aging [43], increase the number of cells positive for SA‐β‐galactosidase staining [44] and induce skeletal muscle atrophy in animal models [45]. In addition, bFGF/FGF2 is a well recognized mitogenic factor that can promote FGFR activation and downstream ERK activation and enhance C2C12 cell proliferation. However, during myogenic differentiation, sustained ERK activation driven by FGF signalling can inhibit terminal differentiation and myotube formation [40, 41, 42, 43, 44, 45, 46]. Consistent with these studies, we observed that D‐gal treatment increased the number of C2C12 cells positive for SA‐β‐galactosidase staining, reduced the formation of myotubes positive for MYHC, decreased MYOG and MYHC expression and increased Atrogin‐1 and MuRF1 expression. These findings indicate that myoblast differentiation was impaired under conditions of injury with senescence features and was accompanied by abnormal changes in molecules associated with atrophy [47]. Notably, D‐gal also increased p‐FGFR1 and p‐ERK levels, suggesting abnormal FGFR1 phosphorylation and ERK activation. BZBS treatment attenuated these abnormal changes and improved impaired myogenic differentiation and abnormal expression of molecules associated with atrophy to varying degrees. After combined treatment with bFGF, p‐FGFR1 and p‐ERK levels increased again and the beneficial effects of BZBS on impaired myogenic differentiation and abnormalities in molecules associated with atrophy were partially weakened. Although p‐FGFR1 and p‐ERK levels were not assessed in vivo in the present study, the in vitro results support the possibility that abnormal FGFR1 phosphorylation and ERK activation may be involved in impaired myoblast differentiation in C2C12 cells exposed to D‐gal. BZBS may act directly on injured myoblasts and improve myogenic differentiation, at least in part, by suppressing these abnormal changes. These findings further suggest that FGFR1/ERK signalling may be involved in the regulatory effects of BZBS on myogenic differentiation. These results provide cellular evidence for the effects of BZBS on myoblast injury characterized by senescence features and are consistent with the senescence‐related changes observed in skeletal muscle from OVX mice.

In the context of skeletal muscle decline associated with postmenopausal oestrogen deficiency, this study used E2 as a positive reference treatment and systematically evaluated the protective effects of BZBS on skeletal muscle at multiple levels, including phenotypic changes, tissue structure and potential molecular mechanisms, rather than relying on a single indicator of efficacy. Notably, the protective effects of BZBS did not appear to depend solely on increasing circulating E2 levels. The present findings support the beneficial effects of BZBS on skeletal muscle under oestrogen deficient conditions, although their clinical relevance to postmenopausal women remains to be established. Based on the abnormalities in molecules associated with senescence observed in skeletal muscle from OVX mice, this study further used a D‐gal‐induced myoblast injury model with senescence features and applied bFGF to counteract the effects of BZBS. The results suggest that BZBS may improve impaired myogenic differentiation and reduce abnormalities in molecules associated with atrophy, with possible involvement of FGFR1/ERK signalling. Collectively, these findings suggest that BZBS ameliorates skeletal muscle impairment associated with oestrogen deficiency and that its effects on myogenic differentiation may involve FGFR1/ERK signalling (Figure 7).

**FIGURE 7:**
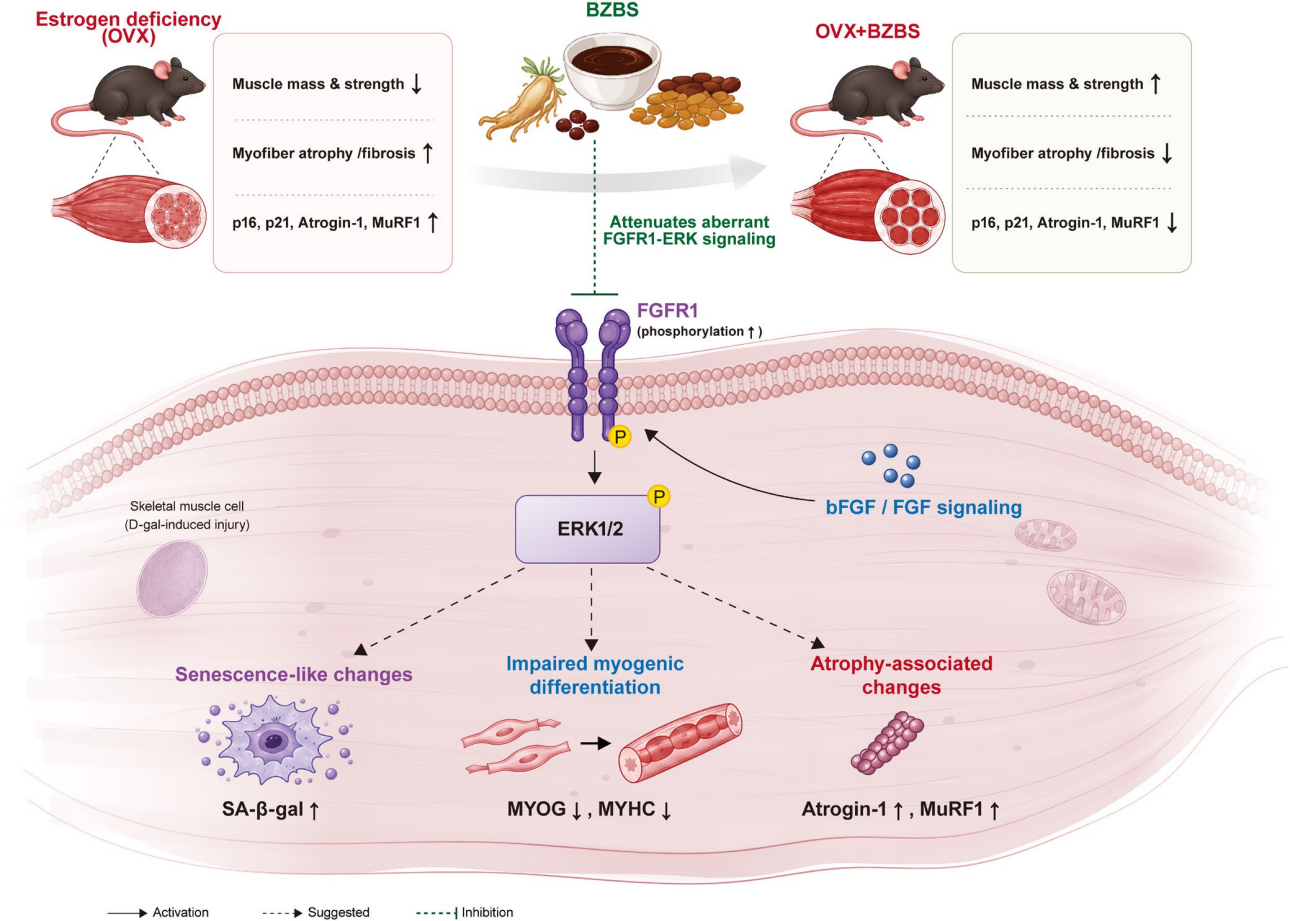
Effects of BZBS on skeletal muscle decline after OVX and the proposed role of FGFR1/ERK signalling. In this study, OVX mice were used to model skeletal muscle changes associated with postmenopausal oestrogen deficiency, with E2 serving as a positive reference treatment. Ovariectomy reduced muscle strength and mass, increased fat accumulation, lowered relative lean mass, decreased metabolic activity during the dark phase and was accompanied by myofiber atrophy and increased expression of markers related to senescence and atrophy. BZBS improved many of these changes, with effects generally similar to those of E2. RNA sequencing, followed by in vitro validation, pointed to the involvement of FGF responses and MAPK signalling. In C2C12 cells exposed to D‐gal, BZBS improved myogenic differentiation and suppressed FGFR1 phosphorylation and ERK activation, whereas bFGF partially weakened these effects. Overall, BZBS alleviated skeletal muscle impairment associated with oestrogen deficiency and FGFR1/ERK signalling may contribute to its effects on myogenic differentiation.

However, several limitations should be considered. First, although the OVX model is widely used to study oestrogen deficiency, it does not fully reproduce the gradual hormonal changes and complex physiological features of natural menopause. Second, some molecular and metabolic analyses were performed with a relatively small sample size, which may limit the statistical power of these findings. Validation in larger cohorts is therefore warranted. Third, although the cell experiments support the involvement of FGFR1/ERK signalling, bFGF is not a specific agonist of FGFR1 and FGFR1 and ERK phosphorylation were not examined in skeletal muscle in vivo. Further studies using more specific approaches are needed to clarify the role of this pathway in the effects of BZBS [48]. Finally, BZBS is a multicomponent herbal formula and the active components responsible for its effects on skeletal muscle and their direct molecular targets remain to be identified.

## 5 Conclusion

In summary, BZBS attenuated skeletal muscle deterioration associated with oestrogen deficiency. BZBS improved impaired myogenic differentiation and reduced molecular changes related to atrophy, while FGFR1/ERK signalling may contribute to these effects. These results suggest that BZBS may help preserve skeletal muscle during oestrogen deficiency.

## Author Contributions

**Dezhi Huang:** conceptualization, investigation, methodology, software, data curation, validation, formal analysis, project administration, visualization, writing – original draft, resources. **Na Xu:** conceptualization, investigation, methodology, validation, software, formal analysis, data curation, resources, project administration, visualization. **Qi Chu:** project administration. **Zhenhua Jia:** conceptualization, methodology, formal analysis, project administration, supervision. **Yan Wang:** conceptualization, formal analysis, methodology, project administration, supervision, funding acquisition. **Jin Xu:** formal analysis, project administration, supervision, funding acquisition, writing – review and editing, conceptualization, methodology.

## Funding

This work was supported by grants from the National Key R&D Program of China (2023YFC3504305, 2021YFC2501700 and 2021YFC2501705), the Shandong Provincial Natural Science Foundation (ZR2023QH350) and the Taishan Scholar Construction Project Special Funding (TS201712092).

## Disclosure

Declaration of Generative AI Use: ChatGPT (GPT‐5.6 Sol, OpenAI) was used to assist with the conceptual design of the schematic in Figure 7. The final figure was created and edited by the authors using vector graphics and Adobe software and all content was reviewed and verified by the authors.

## Conflicts of Interest

The authors declare no conflicts of interest.

## Supporting information


**Table S1:** Primer sequence for qPCR.
**Table S3:** Top 30 GO‐BP enrichment results of the 256 overlapping DEGs.

**Table S2:** Exact *p* values for statistical comparisons.

**Data S1:** Supporting Information.

## Data Availability

The data that support the findings of this study are available from the corresponding author upon reasonable request.
