# Supplementary material for: Bazi Bushen Attenuates Skeletal Muscle Decline Associated With Oestrogen Deficiency and Modulates FGFR1/ERK Signalling
Source: J Cell Mol Med. 2026 Oct 30;30(21):e71407. doi: 10.1111/jcmm.71407 (PMC13721902; doi:10.1111/jcmm.71407)
Supplement: Supplementary file 1 [file JCMM-30-e71407-s003.docx]

**Supplemental Materials and Methods**

#### 1. **Body composition analysis**

Body composition was analyzed in conscious mice using a Minispec LF90 nuclear magnetic resonance (NMR) system (Bruker Optics, Billerica, MA, USA). Lean mass and fat mass were measured without anesthesia.

#### 2. **Metabolic cage analysis**

Metabolic measurements were performed using a Comprehensive Lab Animal Monitoring System (CLAMS; Columbus Instruments, Columbus, OH, USA). Mice were housed individually in metabolic cages for approximately 72 h at an ambient temperature of 21 °C. Spontaneous locomotor activity based on infrared beam breaks, oxygen consumption (VO₂), carbon dioxide production (VCO₂), and energy expenditure (EE) were continuously monitored. VO₂ and VCO₂ were expressed as mL/kg/h, and EE was expressed as kcal/kg/h, with these parameters normalized to body weight. EE was represented by the heat production values recorded by the CLAMS system. The first 24 h were considered an acclimation period and were excluded from analysis. Subsequent metabolic data were analyzed according to the light and dark phases and over a 24 h light-dark cycle. At the end of the experiment, the mice were returned to standard housing cages with free access to food.

#### 3. **Grip strength test**

Forelimb grip strength was measured using a grip strength meter (KW-ZL-1, Nanjing Calvin Biotechnology Co., Ltd., China). During the test, each mouse was placed on a metal grid connected to a force sensor, and the tail was gently pulled backward so that the mouse moved horizontally away from the grid. The maximal force recorded before the mouse released the grid was documented. Each mouse was tested five times with 10 s intervals between measurements. The average of the five peak force values was calculated as the final grip strength and used to evaluate muscle strength.

#### 4. **Histological staining**

Gastrocnemius (GAS) muscle tissues were fixed in 4% paraformaldehyde, embedded in paraffin, and sectioned at 4 μm. The sections were stained using a hematoxylin and eosin staining kit (EE0012, Sparkjade, China) and a Masson trichrome staining kit (Servicebio, Wuhan, China). Images were acquired using a VS200 imaging system (Olympus, Tokyo, Japan). The cross-sectional area of approximately 120 myofibers was measured for each mouse.

#### 5. **Serum hormone measurements**

Serum levels of estradiol (E2), follicle stimulating hormone (FSH), and myostatin were measured using commercial ELISA kits, including a Mouse E2 ELISA kit (JL11790-96T, Jianglai Bio, China), Mouse FSH ELISA kit (JL10239-96T, Jianglai Bio, China), and Mouse Myostatin ELISA kit (JL20385-96T, Jianglai Bio, China). Each serum sample was assayed in duplicate, and absorbance was measured at 450 nm using an ELISA microplate reader (BioTek, USA). Hormone concentrations were calculated from the standard curves, and the mean of the two replicate concentrations was used as the final value for each mouse.

#### 6. **Immunohistochemistry**

Paraffin sections of gastrocnemius muscle tissue were deparaffinized in xylene (10023418) and rehydrated through a graded ethanol series (100092683). Endogenous peroxidase activity was then blocked with 3% H₂O₂ (73113760). These reagents were purchased from Sinopharm Chemical Reagent Co., Ltd. (Shanghai, China). Antigen retrieval was performed by heating the sections in Tris-EDTA buffer (pH 9.0; E9884, Sigma, Germany), followed by blocking with 5% BSA (B2064, Sigma, Germany). The sections were then incubated overnight at 4 °C with primary antibodies against p21 (ab188224, Abcam, UK) and p16 (ab211542, Abcam, UK). The next day, after washing with PBS, the sections were incubated with a secondary antibody conjugated with HRP (WAS1202, Wasci, China) at 37 °C for 30 min. Color development was performed using DAB substrate (BP0770, DAKO, Denmark), and the sections were counterstained with hematoxylin. After dehydration, clearing, and mounting, the sections were observed and imaged under a light microscope (Nikon Eclipse Ci-L, Nikon, Japan). Positive signals appeared as yellow to brown intracellular granular deposits. Brown positive staining areas in the IHC images were segmented by thresholding using ImageJ software, and the positive staining areas of p16 and p21 were quantified.

#### 7. **RNA-seq and bioinformatics analysis**

Total RNA was extracted from gastrocnemius muscle tissue and subjected to quality assessment. mRNA was enriched using Oligo(dT) magnetic beads. The mRNA was then fragmented, reverse transcribed into cDNA using random primers, and used to construct sequencing libraries. After library quality control, sequencing was performed on an Illumina platform. Raw sequencing data were first processed using fastp for quality control. Adapter sequences, reads of low quality, and reads containing poly-N were removed to obtain clean reads. Quality control parameters, including Q20, Q30, and GC content, were then calculated. Clean reads were aligned to the reference genome using HISAT2, and transcript assembly was performed using StringTie. Gene read counts were quantified using featureCounts, and normalized expression levels were expressed as FPKM. Differential expression analysis was performed using DESeq2 based on raw counts. Differentially expressed genes (DEGs) were defined as genes with P value ≤ 0.05 and |log₂FC| ≥ 1. Principal component analysis (PCA), volcano plots, heatmaps, and Venn diagrams were generated based on the DEGs. Gene Ontology (GO) and Kyoto Encyclopedia of Genes and Genomes (KEGG) enrichment analyses of the overlapping DEGs were further performed using clusterProfiler to explore the potential biological functions and signaling pathways associated with transcriptional changes induced by OVX and modified by BZBS treatment.

#### 8. **RNA extraction, cDNA synthesis, and quantitative real-time PCR**

Total RNA was extracted using TRIzol reagent (Invitrogen, 15596-026, USA). RNA concentration and purity were assessed by measuring the OD260/OD280 ratio using a UV spectrophotometer (SHIMADZU, UV-2450, Japan). cDNA was synthesized by reverse transcription using a first strand cDNA synthesis kit (TaKaRa, RR036B, Japan). qPCR was performed using a SYBR Green kit (TaKaRa, RR086B, Japan), with the synthesized cDNA as the template. *Gapdh* was used as the reference gene, and relative mRNA expression was calculated using the 2^−ΔΔCt method. The primer sequences are listed in Supplementary Table S1.

#### 9. **Cell culture and treatment**

Mouse C2C12 myoblasts were purchased from Wuhan Procell Life Science & Technology Co., Ltd. (Wuhan, China). The cells were cultured in DMEM (Gibco, Invitrogen, USA) supplemented with 10% FBS and maintained in a cell culture incubator (150i, Thermo, USA) at 37 °C with 5% CO₂. For differentiation induction, C2C12 cells were seeded in six-well plates at a density of 5 × 10⁵ cells per well. When the cells reached an appropriate confluence, the medium was replaced with DMEM differentiation medium containing 2% horse serum (16050122, Gibco, USA). According to the experimental design, D-gal (20 mg/mL; T0591, TargetMol, USA)^1^, BZBS (100 μg/mL), and/or bFGF (100 ng/mL; 450-33, PeproTech, USA)^2^ were added to the differentiation medium. Cells were cultured for 8 d. D-gal treatment was used to induce a cellular injury phenotype characterized by senescence-associated changes and impaired myogenic differentiation for subsequent mechanistic experiments.

#### 10. **Preparation of BZBS extract**

BZBS ultrafine powder was mixed with 50% ethanol at a ratio of 1:15 (w/v) and extracted by ultrasonication using an ultrasonic processor (SONICS, USA). The extract was then centrifuged at 3500 rpm for 10 min. The supernatant was collected and lyophilized using a freeze dryer (ALPHA 1-2 LD plus, Martin Christ, Germany) to obtain the BZBS extract. The lyophilized extract was dissolved in sterile PBS to prepare a stock solution at a concentration of 250 mg/mL. After sterilization by filtration through a 0.22 μm membrane, the stock solution was diluted with cell culture medium to working concentrations of 20, 100, 200, 500, and 1000 μg/mL for subsequent experiments.

#### 11. **CCK-8 assay for cell viability**

Cell viability was assessed using a CCK-8 kit (KGA317, KeyGEN BioTECH, China) according to the manufacturer’s instructions. Briefly, CCK-8 working solution was added to each well, and the cells were incubated at 37 °C with 5% CO₂ for 1.5 h. Absorbance at 450 nm was then measured using a microplate reader. Cell viability was expressed as the ratio of the OD450 value in each treatment group to that in the control group. Six replicate wells were included for each group, and three independent experiments were performed.

#### 12. **SA-β-galactosidase staining**

Cellular senescence in C2C12 cells was assessed using a senescence-associated β-galactosidase staining kit (KGPAG001, KeyGEN BioTECH, China). After treatment, the cells were fixed and stained according to the manufacturer’s instructions and incubated at 37 °C in the dark. After staining, the cells were observed and imaged under a light microscope. Cells positive for SA-β-galactosidase staining showed blue to green staining.

#### 13. **Immunofluorescence staining**

After treatment, the culture medium was removed, and the cells were washed twice with PBS. The cells were then fixed with 4% paraformaldehyde at room temperature for 30 min and washed three times with PBS for 5 min each. After permeabilization with 0.2% Triton X-100 for 10 min, the cells were washed three times with PBS for 5 min each. The cells were then blocked with 5% BSA at room temperature for 60 min. After blocking, the cells were incubated overnight at 4 °C with a primary antibody against MYHC (sc-376157, Santa Cruz, USA; 1:300) diluted in 5% BSA. The next day, the cells were washed three times with PBS for 5 min each and then incubated with a secondary antibody conjugated with a fluorophore (GB22301, Servicebio, China; 1:100) at room temperature in the dark for 1 h. Finally, the cells were mounted with mounting medium containing DAPI and antifade reagent (S2110, Solarbio, China), and images were acquired using a Nikon laser confocal microscope. The area positive for MYHC was quantified using ImageJ software to evaluate myotube formation.

#### 14. **Western blot analysis**

Skeletal muscle tissue (approximately 30 mg) and cultured cells were washed with ice cold PBS and lysed in RIPA buffer supplemented with phosphatase and protease inhibitors (Epizyme Biomedical, Shanghai, China) on ice. Protein concentration was determined using a BCA Protein Assay Kit (Beyotime, China). Equal amounts of protein were separated by SDS-PAGE and transferred onto PVDF membranes (Millipore, USA). The membranes were blocked with 5% nonfat milk for 1.5 h at room temperature and then incubated overnight at 4 °C with the following primary antibodies: p21 (ab188224, Abcam, UK; 1:1000), p16 (ab211542, Abcam, UK; 1:1000), MuRF1 (55456-1-AP, Proteintech, China; 1:1000), Atrogin-1 (ab168372, Abcam, UK; 1:1000), FGFR1 (YM8066, Immunoway, USA; 1:1000), phospho-FGFR1 (Tyr654) (YP0528, Immunoway, USA; 1:1000), p44/42 MAPK (ERK1/2) (#4695, Cell Signaling Technology, USA; 1:1000), phospho-p44/42 MAPK (ERK1/2) (#5726, Cell Signaling Technology, USA; 1:1000), MYHC (sc-376157, Santa Cruz, USA; 1:1000), MYOG (sc-12732, Santa Cruz, USA; 1:1000), GAPDH (10494-1-AP, Proteintech, China; 1:5000), and β‑actin (66009‑1‑Ig, Proteintech, China; 1:50000). After washing with TBST, the membranes were incubated with a secondary antibody conjugated with HRP (BA1056, Boster, China; 1:5000) for 1 h at room temperature. Protein bands were visualized using an enhanced chemiluminescence detection system, and densitometric analysis was performed using ImageJ software. Total protein levels were normalized to GAPDH, whereas phosphorylated FGFR1 and ERK1/2 levels were normalized to total FGFR1 and total ERK1/2, respectively.

1.	Wang, H.H.*, et al.* Nobiletin Prevents D-Galactose-Induced C2C12 Cell Aging by Improving Mitochondrial Function. *Int J Mol Sci* **23**(2022).

2.	Thiemann, R.F.*, et al.* Regulation of myoepithelial differentiation. *PLoS One* **17**, e0268668 (2022).
